# Biocontrol yeast T‐2 improves the postharvest disease resistance of grape by stimulation of the antioxidant system

**DOI:** 10.1002/fsn3.2940

**Published:** 2022-05-30

**Authors:** Chenyang Wu, Yuci Wang, Dan Ai, Zhuoran Li, Yuanhong Wang

**Affiliations:** ^1^ Tianjin Agricultural University Tianjin China

**Keywords:** antioxidant, biocontrol yeast, grape berries, lipid peroxidation, postharvest resistance, Total phenolics

## Abstract

Table grapes are susceptible to external pathogens during postharvest storage. The resulting continuous oxidative stress causes damage and aging, thereby reducing the defense against disease. In this study, the effect of biocontrol yeast T‐2 on the storage performance of grapes was evaluated. After T‐2 treatment, the grapefruits rot rate and lesion diameter caused by *Botrytis cinerea (B. cinerea)* were significantly decreased at 2–5 days after inoculation (DAI). Additionally, the browning rate and shedding rate of grapefruit during storage were significantly reduced at 2–5 DAI, and the weight loss rate was significantly reduced at 3–5 DAI. The decreased malondialdehyde (MDA) content in grapefruits at 1–5 DAI with T‐2 indicated a reduction in oxidative damage. Furthermore, the activities of antioxidant enzymes such as peroxidase (POD), catalase (CAT), phenylalanin ammonia‐lyase (PAL) were significantly increased during most storage time after being treated with T‐2. Moreover, the contents of total phenolics and flavonoids and the expression levels of key enzyme genes in metabolic pathways were increased after T‐2 treatment during most postharvest storage time, providing evidence that T‐2 changed the biological process of phenolic flavonoid metabolism. The increase in enzymatic and nonenzymatic antioxidants after treatment with T‐2 reflected the strengthening of the antioxidant system, hence postponing fruit senescence and promoting storage performance under the stress of *B. cinerea*.

## Graphical abstract

The effect of biocontrol yeast T‐2 on the postharvest quality and storage performance of grape fruits was tested. The treatment by T‐2 can enhanced the grape fruits resistance to *Botrytis cinerea*, decreased the grape rachis browning index, grape clusters weight loss. T‐2 treatment led to a lower malondialdehyde content and higher activities of antioxidant enzymes. T‐2 treatment increased the content of total phenolics and flavonoids and the expression levels of key genes in related metabolic pathways in grape fruits.

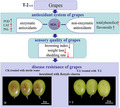

## 1 INTRODUCTION

Grapes are perennial berry plant widely planted in the world. Table grapes are favored by consumers for their unique flavor. However, the maintenance of grapefruit quality after harvest is restricted by internal senescence and the external environment, resulting in limited storage time (Balic et al., 2018; Dong et al., 2020; Ghan et al., 2017). As independent individuals, the postharvest grapefruits will undergo a series of physiological and biochemical changes when dealing with pathogenic bacteria. Reactive oxygen species (ROS) are reactive molecules and free radicals produced by oxygen molecules, and its production is an initial and universal response of plant to pathogen attack (Mittler, 2002; Y. Xu et al., 2019). Uncontrolled over‐production of ROS disrupts normal metabolism by oxidizing proteins and lipids affecting the integrity of cell membranes and inactivating cellular functions (Xu et al., 2008). The gray mold caused by *Botrytis cinerea* (*B. cinerea*) is one of the most widely postharvest diseases in grapes. Considerable researches show that *B. cinerea* can initiate the ROS stress on plants and easily lead to lipid peroxidation which is represented by malondialdehyde (MDA) content, and considered to be the main feature of harvested fruits undergoing cell damage and senescence (Deighton et al., 1999; Temme & Tudzynski, 2009). However, senescence and death of plant cells are conducive to pathogen infection, leading to host susceptibility (Laxa et al., 2019; Mengiste, 2012). Therefore, postponing tissue aging is of great significance for maintaining the quality of grapes after harvesting and reducing the occurrence of disease (Rahman et al., 2020).

To adapt to resist the invasion of external pathogens, the content of secondary metabolites will be adjusted to maintain the life activities of fruits during postharvest storage (Kim et al., 2011; Xu et al., 2021). Among them, the substances of phenolics and flavonoids, as the main active antioxidant substances in grapes, play an important role in the coping with oxidative stress. Therefore, keeping a high concentration of phenolics and flavonoids after grape harvest will improve grape preservation and storage (Georgiev et al., 2014; Singh et al., 2019; Thompson et al., 1987). The content of these secondary metabolites in fruits depends on different factors (Li et al., 2019). Recent studies have shown that plant defense induced by biocontrol bacteria is one of the most effective strategies in enhancing the production of secondary metabolic compounds (Houille et al., 2015; Romero et al., 2020). For example, combined using both *Bulgaricus Strain* F17 and *Leuconostoc lactis Strain* H52 can increase the content of phenolic substances in grapes, strengthen the antioxidant capacity of the fruits, delay the ripening and aging process of grapes, and prolong the storage time of grapes after harvest (Ramirez‐Estrada et al., 2016). Godana found that *Pichia Anomala* increased the accumulation of total phenols and flavonoids in blueberries and increased resistance to gray mold (Godana et al., 2021).

Besides metabolites, antioxidant enzymes are also an important part of the antioxidant system. Plants can launch an arrangement of antioxidant enzymes to hunt destructive ROS and defend cells from oxidative injury under persistent oxidative stress (Luo, Li, et al., 2015; Singh et al., 2019). One of the emerging strategies to mitigate loss during storage is to apply natural inducers to stimulate the response of fruits and increase the content of antioxidant enzymes. Zhou found that *Bacillus amyloliquefaciens* NCPSJ7 can reduce the content of antioxidant enzymes such as peroxidase (POD) and polyphenol oxidase (PPO), and control the occurrence of gray mold (Zhou et al., 2020). Zhao found that *B. subtilis* CF‐3 can reduce plant cell damage by activating antioxidant enzymes such as POD and catalase (CAT) (Zhao et al., 2019).

In recent years, most researches on yeast‐mediated fruits decay control focuses on the induction of defensive enzymes against fungal pathogens (Castoria et al., 2003; Pedro et al., 2019; Zhang et al., 2017). However, there is also evidence that yeast‐induced antioxidants can resist the damage caused by oxidative stress induced by pathogens in postharvest fruits. Xu proposed that the increase in antioxidants can be used as a scavenger to reduce the pathogen‐induced oxidative damage, ensuring resistance of fruits to pathogens, and using antagonistic yeast (*Pichia membranaefaciens*, *Cryptococcus laurentii*, *Candida guilliermondii,* and *Rhodotorula glutinis*) to verify on peaches (Xu et al., 2008). Li showed in the study that the POD activity of apples inoculated with *Rhodosia glutinosa* was higher than that of the control, indicating that the cell lipid peroxidation (MDA content) was lower than that of the control (Li et al., 2011). Hershkovitz found that the application of *Metschnikowia fructicola* increased the activity of phenylalanin ammonia‐lyase (PAL) and the expression of genes related to the synthesis of phenolics compounds in a variety of plant defense metabolites in the phenylpropanoid biosynthesis metabolic pathway increased (Hershkovitz et al., 2012). Chen pointed out that genes encoding phenylpropane biosynthesis‐related enzymes in citrus fruits, such as *PAL*, were upregulated and showed higher levels of total phenolic compounds when treated with *Pichia galeiformis*, including flavonoids, ferulic, and erucic acid indicated that *P. galeiformis* can induce the accumulation of resistant metabolites by modulating the postharvest citrus phenylpropanoid biosynthesis pathway. Based on their research results, it was hypothesized that yeast can reduce oxidative damage caused by pathogenic bacteria by strengthening the oxidation system, including non‐enzymatic and enzymatic antioxidants (Chen et al., 2021).

Yeasts from the *Metschnikowia Pulcherrima* (*M. Pulcherrima*) branch of the genus *Macro Ascomycetes* have antagonistic effects on many types of microorganisms (Sipiczki, 2020). Studies have shown that *M. Pulcherrima* has a control effect on various postharvest fruits disease, but there is still a lack of relevant researches on postharvest grapes (Oro et al., 2018; Tian et al., 2018). The *M. Pulcherrima* T‐2 (T‐2), which was screened in the laboratory and has a control effect on gray mold, was applied to grapes inoculated with *B. cinerea*. In this study, we found that T‐2 reduced the grapes rot caused by *B. cinerea* during the postharvest. More importantly, T‐2 can maintain better sensory quality of grapes and delay fruits senescence. Then we evaluated the MDA content after T‐2 treatment, the main indicator of oxidative damage in grape berries, on the basis of its significant reduction, we evaluated the changes of antioxidant enzymes including CAT, PAL, and POD, biologically active substances flavonoids and total phenolics in the antioxidant system. In addition, the expression levels of genes related to phenylalanine metabolism and total phenolics were also measured in fruits after T‐2 treatment. Through the above biochemical and molecular analysis, we wonder whether the fresh‐keeping performance with the T‐2 treatment may be attributed to the activation of intrinsic antioxidant capacity.

## 2 MATERIALS AND METHODS

### 2.1 Biological materials and experimental design

“Shine Muscat” grapes were harvested at commercial maturity from grape plantation located in Jinghai, Tianjin, China. The grape clusters with the same maturity and without mechanical damage, disease, insect pests, are transported to the laboratory immediately after being picked.

Biocontrol yeast: *Metschnikowia Pulcherrima* T‐2 was provided by the Laboratory of Plant Immunity and Biological Control, Tianjin Agricultural University, China. A single colony was cultured in YPD solution at 180 r/min and centrifuged at 28°C, the supernatant was discarded, washed with sterile water, and obtain T‐2 cells. Diluting was performed with sterile water and counted 1 × 10^8^ CFU/ml on a hemocytometer.

*Botrytis cinerea* (*B. cinerea*): Provided by the Laboratory of Plant Immunity and Biological Control, Tianjin Agricultural College, China. Take it out a week before use and activate it in PDA medium. Transfer to a PDA plate and culture for 10 days until spores are formed. After being washed with sterile water, four layers of sterilized cheesecloth are filtered to obtain the spore liquid, which is counted to 1 × 10^6^ CFU/ml with a hemocytometer.

Disease resistance test of grapefruits: Select uniform in size and relatively consistent in maturity, cut them with sterile scissors at 3 mm of the stalk, and immerse them in 70% (v/v) ethanol soaking for 30s, 2% (v/v) sodium hypochlorite soaking for 2 min, washed with tap water, and put them in the sterile typhoon for 1 h to dry the surface moisture before use. Use a cork borer to punch holes at the equator (diameter 2 mm, depth 5 mm). For the treatment group, each well was inoculated with 20 μl of bacterial suspension T‐2, while 20 μl of sterile water was used for the control group, after 2 h of inoculation with 20 μl of *B. cinerea* spores of all berries, then grape berries are stored for 5 days at a humidity of 85%–90% and a temperature of 25°C. Each treatment consists of three replicates, each with 165 grape berries, of which 15 are used to determine the diameter of the disease spot and the decay rate in 1–5 days, and 30 fruits are taken out every day in 1–5 days, 15 fruits were ground and mixed with liquid nitrogen, of which are used for both of determination of flavonoids, total phenolics and extraction of RNA extraction, 15 fruits were used for the determination of malondialdehyde (MDA) content and antioxidant enzyme activity.

Validation of T‐2 on grapefruits preservation effect: Select fresh grape clusters that are healthy, disease‐free, no fruit shedding, and relatively consistent maturity. The treatment group was soaked and inoculated with 1 × 10^8^ CFU/ml T‐2 bacterial suspension, and the control group was soaked and inoculated with sterile water. After 2 h, each cluster of grapes was soaked and inoculated with a concentration of 1 × 10^6^ spore/ml *B. cinerea* spore suspension. After processing, the grape clusters are stored for 5 days at a humidity of 85%–90% and a temperature of 25°C. Each treatment consists of three replicates with six grape clusters per replicate. The browning rate of fruits shaft, the rate of fruits removal, and the rate of weight loss are measured every day.

### 2.2 Quality evaluation of grapes

Weight loss: Rotting grapes were counted daily from 0 to 5 days after treatment, and the results are indicated as percentage (%).

Browning index: Evaluate the browning index of the grape rachis according to the browning grade reference Ni et al. (2016). The browning index is calculated by the following formula every day, where d is the grape shaft for scale browning; ƒ is their respective number; N is the total number of grape shafts examined; and D is the highest degree of scale browning.

(1) $$\text{Browning index}\;\left(\%\right)=\left[\sum \left(\mathrm{df}\right)/\mathrm{ND}\right]\times 100$$

Fruit shedding rate was calculated as per the following formula:

(2) $$\text{Shedding rate}\;\left(\%\right)=\left[\sum \text{shed fruit weight}/\text{grape cluster weight}\right]\times 100\left(\%\right)$$

weight loss: Each grape string was weighed after each sampling date from 0 to 5 days after treatment, and weight loss is indicated as a percentage (%).

(3) $$\text{Weight loss}\;\left(\%\right)=\left[\text{Initial fruit weight}-\text{fruit weight after storage}/\text{Initial fruit weight}\right]\times 100\left(\%\right)$$

### 2.3 Content of MDA

Lipid peroxidation was determined in terms of MDA content by TBA reaction, The samples of fruits were ground into powder in liquid nitrogen, 2 g was used for the determination of MDA content. The determination refers to the method of Luo (Luo, Wan, et al., 2015), and the final result is expressed in nmol/g FW.

### 2.4 Activity of antioxidant enzymes

The samples of fruits were ground into powder in liquid nitrogen, 3 g of them were used to determine antioxidant enzyme activity, and 6 ml sodium phosphate buffer (pH 6.0) was added. The crushed fruit was then centrifuged at 8500 g/min for 10 minutes and collected the supernatant for the enzymatic activity assay. POD was determined reference to the method of Li et al. (2011); PAL and CAT were determined according to the method of Javadi Khederi et al. (2018). Results were obtained as 1 POD PAL, CAT active unit (U) when the absorbance value of the reaction system increased by 0.01 per min with FW, and the results are shown as U/g FW.

### 2.5 Determination of the content of flavonoids and total phenolic

The grape berry samples in the previous experiment were stored, cut into small pieces, stored at ‐ 80°C, and ground in liquid nitrogen during the experiment to measure the content of flavonoids and total phenolic. The total flavonoids were determined as described by Yuan et al. (2019). Total phenolic content was measured using the Folin–Ciocalteu method and detected at a wavelength of 760 nm using a spectrophotometer (Fu et al., 2016).

### 2.6 RNA isolation and quantitative real‐time PCR

In the previous storage experiment, grape berry samples were taken daily, cut into small pieces, liquid nitrogen ground, and stored at −80°C to extract for RNA extraction. Total RNA extraction from grapefruit samples was carried out according to the instructions of the TIANGEN RNA prep Pure Polysaccharide Polyphenol Plant Total RNA Extraction Kit. Using total RNA as a template, Takara Prime Script TM RT regent Kit with gDNA Eraser (Perfect Real‐Time) was used for reverse transcription and purification. *Vv‐Actin* gene was selected as an internal reference gene; Primer sequences of the *Vv‐PAL, Vv‐C4H, Vv‐4CL, Vv‐CHS*, and *Vv‐Actin* genes are shown in Table 1, which were designed and completed by Sangon Bioengineering, Shanghai, China. Quantitative real‐time PCR (qRT‐PCR) was performed to estimate the relative expression of four selected genes. The primers and reaction system are added according to the instructions (Takara).

**TABLE 1:** The primer sequences of flavonoid and phenolic synthesis pathway genes analyzed in this study

Gene		Primer sequence
*Vv‐PAL*	*Vv‐phenylalanin ammonia‐lyase*	F 5′ CAACCAAGATGTGAACTCCTT 3′
		R 5′ TTCTCCTCCAAATGCCTC 3′
*Vv‐C4H*	*Vv‐Cinnamate‐4‐hydroxylase*	F 5′ AAAGGGTGGGCAGTTGAGTT 3′
		R 5′ GGGGGGTGAAAGGAAGATAT 3′
*Vv‐4CL*	*Vv‐4‐coumarate coaligase*	F 5′ CGAAGAACCCGATGGTGGAGA 3′
		R 5′ CACGAGCCGGACTTAGTAGGA 3′
*Vv‐CHS*	*Vv‐Chalcone synthetase*	F 5′ CGACACGTCTTGAGCGAGTATGG 3′
		R 5′ TCAGCCGACTTCCTCCTCATC 3′
*Vv‐Actin*	*Vv‐Actin*	F 5′ CTTGCATCCCTCAGCACCTT 3′
		R 5′ TCCTGTGGACAATGGATGGA 3′

## 3 RESULTS

### 3.1 T‐2 reduce gray Mold in grapes

**FIGURE 1:**
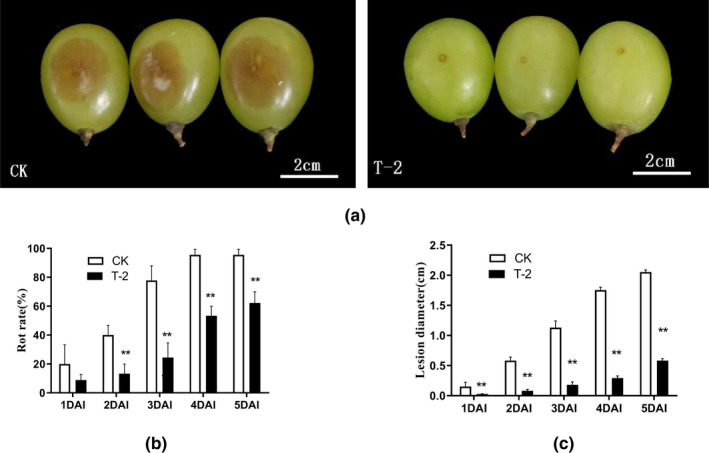
Effect of T‐2 on fruit decay during storage after grape harvest. The surface of the grapefruit was disinfected and pretreated and then inoculated in groups. Grape berries were first inoculated with the T‐2 treatment group (T‐2) and sterile water (CK), then inoculated with *B. cinerea*. Measured after inoculation at 25°C for 5d (a) the decay rate of grape berries caused by B. cinerea at 3 DAI (days after inoculation). Photographs depicting representative disease development. From left to right, CK and T‐2 treatment group. (b) the rate of rot grapefruit caused by B. cinerea at 1–5 DAI. The horizontal axis is the storage days after inoculation, and the vertical axis is the percentage of rotten fruit (%). (c) the lesion diameter of grapefruit caused by B. cinerea at 1–5 DAI. The horizontal axis is the storage days after inoculation, and the vertical axis is the diameter of the lesion (cm). The error line is the standard deviation of multiple biological replicates; the results are mean ± standard deviation and the analysis of the significance of the data differences was performed using the *t*‐test; *p < .05; **p < .01

Gray mold caused by Botrytis cinerea (*B. cinerea*) is the main disease in the postharvest storage of grapes (De Simone et al., 2020). In this study, grape berries were first inoculated with the T‐2 treatment group (T‐2) and sterile water (CK), then inoculated with *B. cinerea*. After infected grape berries with *B. cinerea*, the lesions began to appear on the surface and showed watery spots, partially recessed, and the color of the depression gradually changed to dark brown, gray‐white hyphae appear, the picture showed the state of the disease of the grapes on 3 DAI (days after inoculation) (Figure 1a). The rot rate of the grapes in the T‐2 treatment group was significantly lower than that of the CK at 2–5 DAI, which was 96% in the CK and only 62% in the T‐2 treatment group at 5 DAI storage (Figure 1b). The lesions diameter on the surface of grape berries was also significantly lower than that in the CK group (Figure 1c).

### 3.2 T‐2 maintains the sensory quality of grapes

**FIGURE 2:**
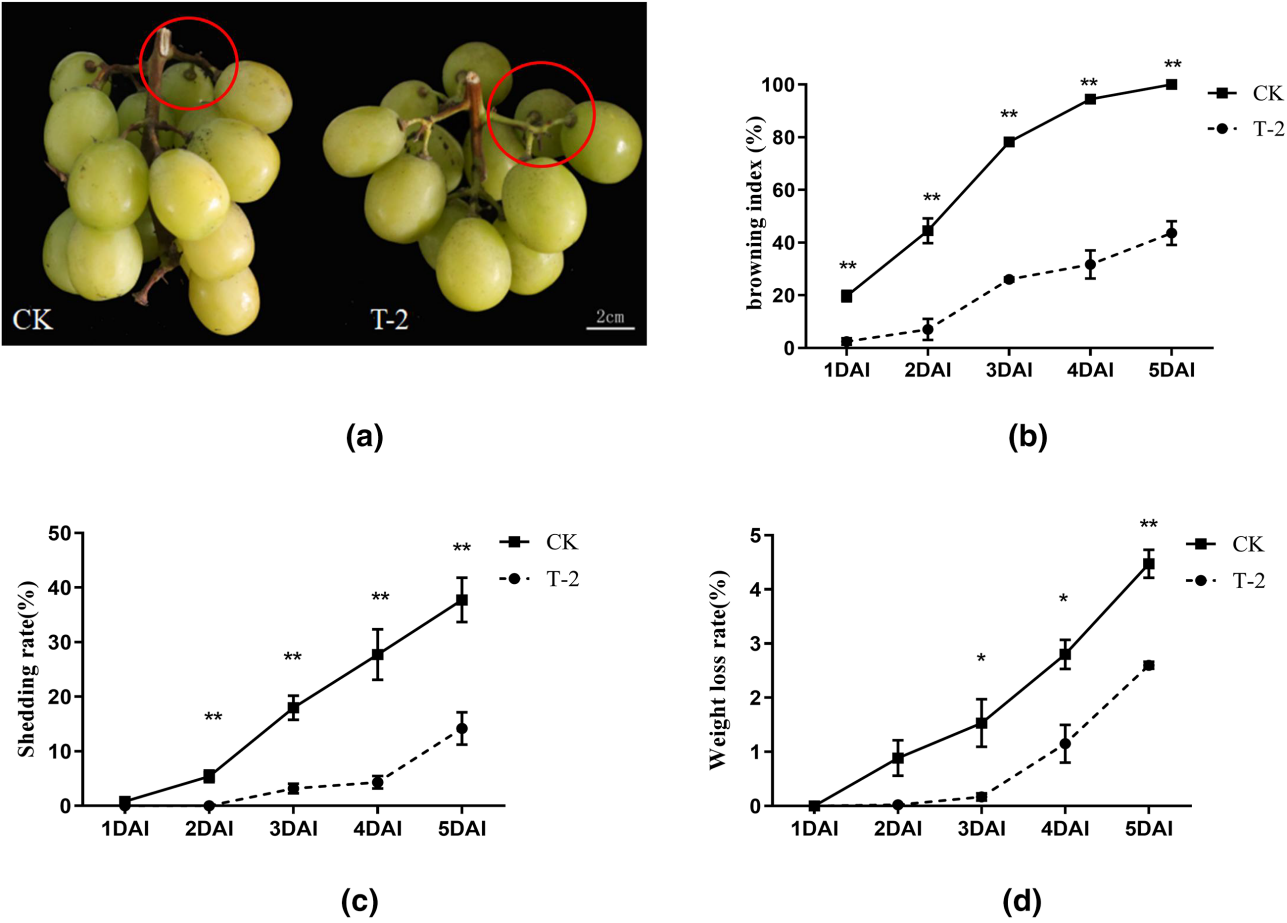
Effects of T‐2 treatment on the sensory quality of grapes. (a) Comparison of browning of the grape rachis at 3 DAI of T‐2 treatment groups. From left to right, CK and T‐2 treatment group. (T‐2) and control groups (CK). (b) Changes in browning index between 1 and 5 days. The horizontal axis is the storage days after inoculation, the vertical axis is browning index (%). (c) Shedding rate of grape clusters. The horizontal axis is the storage days after inoculation, the vertical axis is shedding rate (%) (d) weight loss percentage of grape berries. The horizontal axis is the storage days after inoculation, the vertical axis is weight loss rate (%). The error line is the standard deviation of multiple biological replicates; the results are mean ± standard deviation and the analysis of the significance of the data differences was performed using the *t*‐test; *p < .05; **p < .01

Grape berry senescence is usually accompanied by physiological and biochemical changes, which is manifested by the decrease in sensory quality after harvest. T‐2 can delay the browning index of the grape rachis, the weight loss rate of the grape clusters, and the rate of the grapefruit shedding. Grape richis browning is a common problem that affects grape quality and consumer preferences. The T‐2 treated grape rachis still maintained a green appearance at 3 DAI (days after inoculation), while the control (CK) clusters showed severe browning (Figure 2a). The browning index of the T‐2 treatment group (T‐2) remained significantly different from that of CK at 1–5 DAI, with a decrease of 56% in the T‐2 treatment group compared to CK at 5DAI (Figure 2b). And after T‐2 treatment, the grape berry shedding rate was significantly reduced compared to CK at 2–5 DAI, with a 62% decrease in the T‐2 treatment group compared to CK at 5DAI (Figure 2c). Weight loss rate is one of the important indicators to measure the aging degree of grapes. The weight loss rate of grape groups after T‐2 treatment was significantly lower than that of CK in 3–5 DAI, with a 42% decrease in the T‐2 treatment group compared to CK at 5 DAI (Figure 2d).

### 3.3 T‐2 reduce the content of malondialdehyde (MDA) in grapes

**FIGURE 3:**
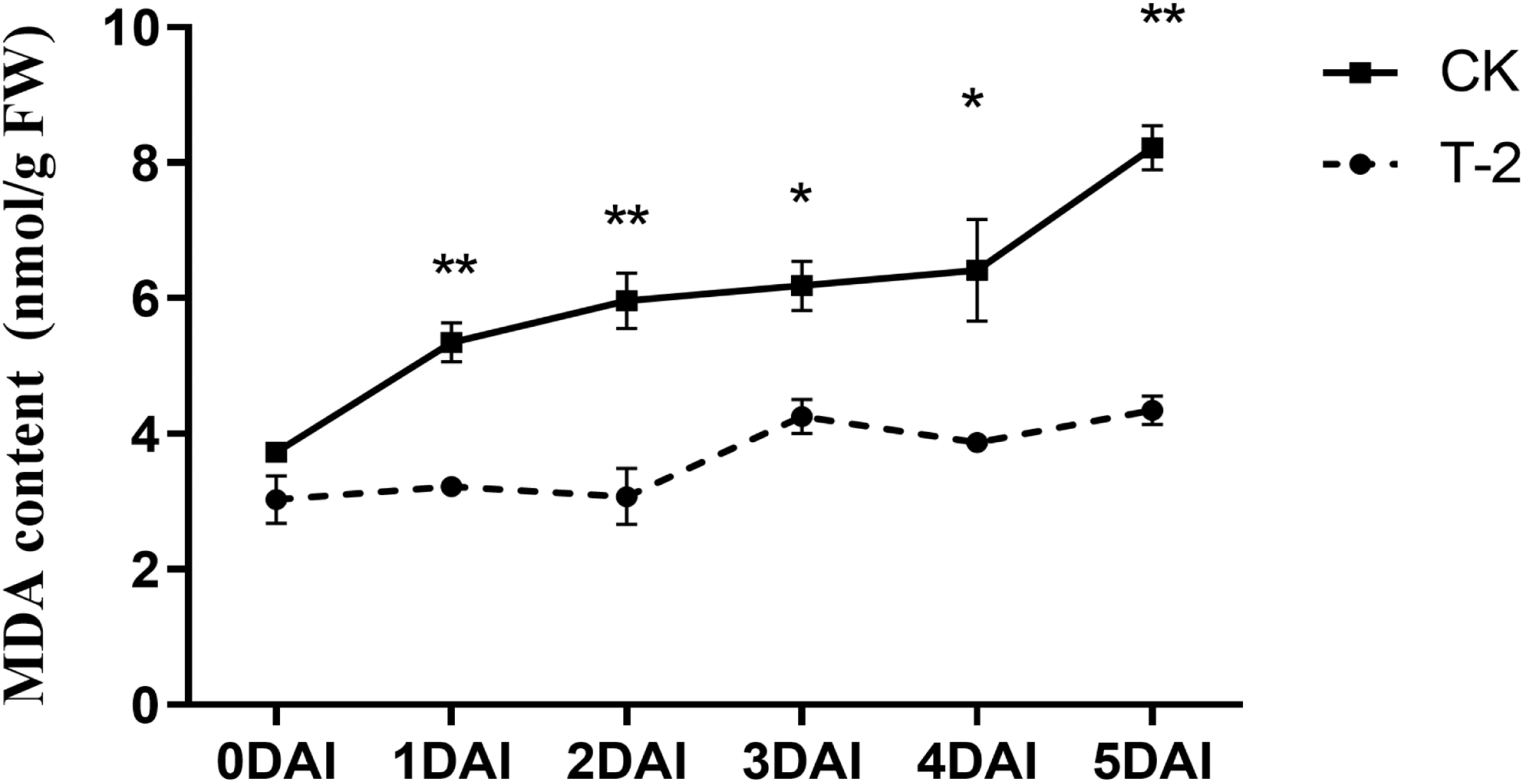
Effect of T‐2 on the content of MDA in grape berries. Both groups were inoculated with B. cinerea, 2 h later inoculated with T‐2 as the treatment group (T‐2), and inoculated with the same dose of sterile water as the control group (CK). The horizontal axis is the storage days after inoculation, the vertical axis is content of MDA (nmol/g FW). Samples were measured separately at 1–5 DAI. The error line is the standard deviation of multiple biological replicates; results are mean ± standard deviation and the analysis of the significance of the data differences was performed using the *t*‐test; *p < .05; **p < .01

The lipid peroxidation content is the main factor that accelerates browning and is considered to be the main feature of harvested fruits that undergo cell damage and senescence. Lipid peroxidation in fruits is usually represented by MDA content (Deighton et al., 1999; Dongdong Li et al., 2017). In this experiment, the MDA content in grape berries was measured separately at 1–5 DAI, and it was found that after inoculation with *B. cinerea*, the MDA content in the fruit began to increase and the MDA level of the control group (CK) increased rapidly at 1 DAI. In the T‐2 treatment group (T‐2), the level of MDA in the fruit was significantly lower than that of CK at 1–5 DAI and the difference was the largest at 5 DAI, which was 8.3 nmol/gFW in the CK and only 4.3 nmol/gFW in the T‐2 group (Figure 3).

### 3.4 T‐2 stimulates activities of antioxidant enzymes

**FIGURE 4:**
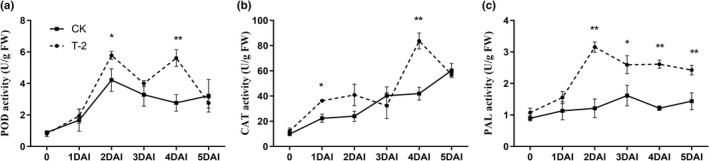
Effect of T‐2 on antioxidant enzymes in grape berries. (a) Changes in peroxidase (POD) activity. The horizontal axis is the storage days after inoculation, the vertical axis is activity of POD (U/g FW). (b) Changes in catalase (CAT) activity. The horizontal axis is the storage days after inoculation, the vertical axis is the activity of CAT (U/g FW). (c) Changes in phenylalnine ammonia‐lyase (PAL) activity. The horizontal axis is the storage days after inoculation, the vertical axis is activity of PAL (U/g FW) samples that were measured separately at 1 to 5 DAI. The error line is the standard deviation of multiple biological replicates; results are mean ± standard deviation and the analysis of the significance of the data differences was performed using the *t*‐test; *p < .05; **p < .01

Antioxidant enzyme is an important part of the grape antioxidant system, which has the function of resisting oxidative damage (Li et al., 2016). The activity of antioxidant enzymes (POD, CAT, and PAL) of grape berries was determined during storage. The POD of all treated fruits increased significantly at 2 DAI and then the POD activity of CK started to decrease. The POD activity of the T‐2 treated group (T‐2) was higher than that of CK at 2–4 DAI and was significantly different from CK at 2 DAI, 4 DAI, and reached 2.0 times CK at 4 DAI (Figure 4a). The CAT activity showed an overall upward trend in all fruits treated. The CAT activity of T‐2 group was higher than CK at 1–2 DAI and 4 DAI, and was different from CK at 1 DAI and 4 DAI, which constitutes a significant difference with the group reaching 2.0‐fold of CK at 4 DAI (Figure 4b). After inoculation treatment, the PAL activity of the fruit treated with T‐2 and CK had a great difference. The PAL activity in the T‐2 group was significantly higher than that of CK at 2–5 DAI, and reached a peak at 2 DAI, which was 2.6 times the changes of CK (Figure 4c).

### 3.5 T‐2 increases the contents of flavonoids, total phenolics in grapes

**FIGURE 5:**
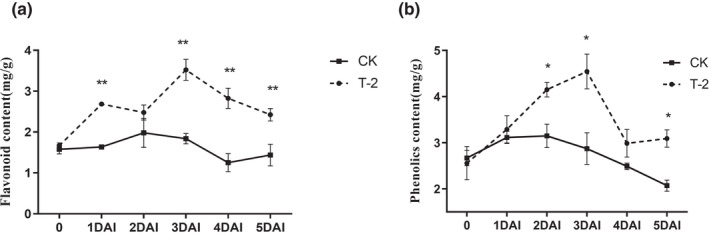
Effect of T‐2 on the contents of flavonoids, total phenolics in grape berries. (a) Flavonoids content in grape berries. The horizontal axis is the storage days after inoculation, the vertical axis is the contents of flavonoids (mg/g). (b) Total phenolics content in grape berries. Samples were measured separately at 1 to 5 DAI. The horizontal axis is the storage days after inoculation, the vertical axis is the contents of total phenolics (mg/g). The error line is the standard deviation of multiple biological replicates; results are mean ± standard deviation and the analysis of the significance of the data differences was performed using the *t*‐test; *p < .05; **p < .01

Secondary metabolites in plants play an important role in resisting oxidative damage. Secondary metabolites in plants play an important role in resisting oxidative damage. In combination with the phenylpropane metabolic pathway (the synthesis pathway of flavonoids and phenolics), the key rate‐limiting enzyme PAL is activated, and the content of flavonoids and total phenolics is determined. The quantitative results showed that the content of flavonoids and total phenolics in the T‐2 treatment group (T‐2) was always higher than that of the control group (CK). The content of flavonoids in the T‐2 group increased significantly and was significantly higher than that of CK in 1 DAI, which can reach 1.5‐fold than that of CK, and the content of flavonoids continues to increase in 3 DAI to reach the maximum value, and has remained significantly higher than the level of CK level (Figure 5a). The total phenolics content of the T‐2 group always remained higher than the level of CK during the observed storage period. The total phenolics concentration of the T‐2 treatment reached its peak value at 3 DAI, which can reach 1.9‐fold than that of CK, then decreased thereafter at 4 DAI, which was significantly higher than CK (Figure 5b).

### 3.6 T‐2 induce expression of the phenylpropanoid pathway genes in grapes

**FIGURE 6:**
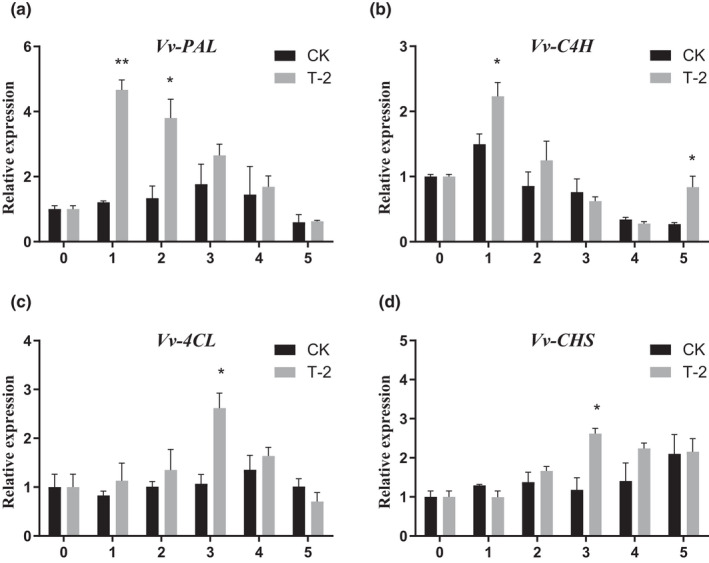
Effect of T‐2 on the expression of genes of the phenylpropanoid pathway in grapes. (a) Relative gene expression levels of *Vv‐phenylpropanoid ammonia‐lyase* (*Vv‐PAL*). The horizontal axis is the storage days after inoculation, the vertical axis is the expression levels of Vv‐PAL. (b) Relative gene expression levels of Vv‐cinnamate‐4‐hydroxylase (Vv‐C4H). The horizontal axis is the storage days after inoculation, the vertical axis is the expression levels of Vv‐C4H. (c) Relative gene expression levels of Vv‐4‐coumarate coaligase (Vv‐4CL). The horizontal axis is the storage days after inoculation, the vertical axis is the expression levels of Vv‐4CL. (d) Relative gene expression levels of Vv‐chalcone synthase (Vv‐CHS). The horizontal axis is the storage days after inoculation, the vertical axis is the expression levels of Vv‐CHS. Samples were measured separately at 1 to 5 DAI. The error line is the standard deviation of multiple biological replicates; results are mean ± standard deviation and the analysis of the significance of the data differences was performed using the *t*‐test; *p < .05; **p < .01

In the experiment of determination of enzyme activity, the rate‐limiting enzyme of the PAL phenylpropanoid pathway in fruits treated with T‐2 increased significantly. Therefore, the expression patterns of genes encoding phenylpropanoid ammonia‐lyase (PAL), cinnamate‐4‐hydroxylase (C4H), 4‐coumarate coaligase (4CL), chalcone synthase (CHS) which in the phenylpropanoid pathway under T‐2 treatment were evaluated. After T‐2 treatment, the relative expression of *PAL* in the fruit increased rapidly on the first day, reached a 3.85‐fold change difference of CK, and formed a very significant difference from the control group (CK), and the expression gradually decreased in the later period, but was still higher than the control. The expression of *C4H* in the fruit has a similar pattern to that of *PAL*, T‐2 treatment expression reached 1.49‐fold change difference of CK at 1 DAI (Figure 6a,b). The expression patterns of *4CL* and *CHS* were similar, and the general trend showed a slow upward trend at an early stage. The rapid increase in expression of the T‐2 treatment group was detected at 4 DAI, in which expression levels were 2.46‐ and 2.23‐fold change significantly different from that of the control CK (Figure 6c,d).

## 4 DISCUSSION

Due to external biological stress, the quality of table grapes decreased rapidly. In production, chemical agents are used to fight fungi and maintain the quality of grapes. However, chemical agents will pose a great threat to the environment (Dias et al., 2020; Konuk Takma & Korel, 2017). Here, we provide a safe and effective alternative to maintain the postharvest performance of grape berries by making full use of biological bacteria T‐2. In this study, treatment with T‐2 and then inoculation with *B. cinerea*, the rot rate was reduced and lesion diameter was inhibited (Figure 1). More importantly, the grape berries of the treatment group maintained good sensory quality. For example, the grape rachis browning index, the weight loss rate, and the shedding rate of the T‐2 treatment were than that of the control group (CK) (Figure 2). The destruction of the cell structure caused by oxidative damage is an important factor leading to dehydration, an important reason for aging and weight loss. The browning of the grape rachis is often caused by the destruction of the cell membrane structure suffered from lipid oxidization (Carvajal‐Millan et al., 2001). T‐2 reduced the weight loss rate of grape berries and the browning index of the grape rachis. This suggested that T‐2 may play a positive role in strengthening the antioxidant system to resist oxidative damage from pathogens.

The antioxidant system, made up of a series of antioxidants and antioxidant enzymes, protects plants from oxidative damage (Singh et al., 2019). In postharvest systems, MDA, as a product of membrane lipid peroxidation, reflects the intensity of cell membrane peroxidation, and the increase in lipid peroxidation is considered to be the main characteristic of plant oxidative damage and senescence (Aghdam et al., 2020; Ayala et al., 2014). Among them, the increased activity of catalase (CAT) and peroxidase (POD) can inhibit the accumulation of ROS intermediate oxygen intermediates (ROI) (Wagner et al., 2013), thus reducing lipid peroxidation (MDA content) caused by the continuous accumulation of ROI (Borsani et al., 2001). In this study, T‐2 increased the activity of POD and CAT in grapes (Figure 4a,b). This result is consistent with the research results of that the biocontrol bacteria NCPSJ7 activating the relevant defense enzymes in grapes (Zhou et al., 2020). Subsequently, Li found that four antagonistic yeasts can increase oxidase levels on apples (Li et al., 2011). It highly inhibited MDA content and reduces oxidative damage. The same effect was observed when used *Hanseniaspora uvarum* yeast on strawberries (Wang et al., 2019). Phenylalanin ammonia‐lyase (PAL) is related to secondary metabolism in plants (Luo et al., 2008). PAL is a key and rate‐limiting enzyme in the metabolic pathway of phenylpropane, which affects the synthesis of bioactive substances of phenolic and flavonoids in plants (Chen et al., 2006). Studies show that the activation of PAL can lead to a decrease of MDA content (Chen et al., 2019). In this experiment, the activity of PAL in the fruit after T‐2 treatment remained significantly higher than that of CK throughout the storage process. Combined with the lower MDA content caused by T‐2 (Figure 3), it was speculated that T‐2 reduced the membrane lipid peroxidation of grapefruits by increasing antioxidant enzymes, and reduced the oxidative damage caused by oxidative stress in grapes.

The content of flavonoids and total phenolics are key quality parameters for evaluating the storage effect of table grapes. These secondary metabolites help to maintain a balanced metabolism by quenching ROS and also play a direct antibacterial effect in plants (Apel & Hirt, 2004; Kumar Patel et al., 2020). Zheng's research showed that activation of PAL in plants increased the total phenolic content, strengthened the antioxidant level of pear fruits, and significantly reduced the MDA content (Zheng et al., 2019). The research results of Chen and Manquian also supported this conclusion (Chen et al., 2019; Manquian‐Cerda et al., 2018). After treatment with T‐2, the content of flavonoids and total phenolics was significantly higher than those of the control group (Figure 5). We interpret these results as the effect of PAL activity (Figure 4c). In the study by Godana, it was found that the yeast *Pichia anomala* also had the same effect of activating the activity of the PAL and increasing the content of flavonoids in grapes, which described the role of flavonoids and total phenolics in defense against pathogens (Godana et al., 2021). The expression levels of the key enzyme‐encoding genes of the phenolics metabolism pathway in grapes were further determined. The expression of the *Vv‐phenylpropanoid ammonia lyase* (*Vv‐PAL*), *Vv‐cinnamate‐4‐hydroxylase* (*Vv‐C4H*), *Vv‐4‐coumarate coaligase* (*Vv‐4CL*), and *Vv‐chalcone synthase* (*Vv‐CHS*) genes (Figure 6) increased during T‐2 treatment, the content of phenolic acids was positively correlated with the expression of *Vv‐C4H* and *Vv‐CHS*, which was involved downstream of the flavonoids pathway (Conde et al., 2016; Thiruvengadam et al., 2015). The expression level of genes related to secondary metabolism indicates that T‐2 can affect the accumulation of flavonoids and total phenolics in fruits. We believe that in this experiment flavonoids and total phenolics acted as antioxidants and bacteriostatic agents, to delay grape senescence and strengthen the grape's defense system, which is consistent with the results of the reduction in grape decay rate and lesion diameter (Figure 1). By summarizing the results of the increase in the content of non‐enzymatic antioxidants (flavonoids and total phenolics compounds) and antioxidant enzymes (CAT, POD, PAL) in fruits treated with T‐2, this study indicates that strengthening the oxidation system to reduce oxidative damage may be the T‐2 biocontrol mechanism, which is of great significance for ensuring cytomembrane integrity and slowing down aging, thereby reducing the invasion of *B. cinerea* and fruit rot after fruit harvest.

## 5 CONCLUSIONS

T‐2 increased the activity of antioxidant enzymes (POD, CAT, PAL) and the content of antioxidant secondary metabolites (flavonoids and phenolics compounds) of grape berries. Whereas reduced the accumulation of MDA. Taken together, T‐2 plays an important role in reducing the oxidative damage on grape berries and inducing antioxidant defense response to strengthen the resistance against fungal pathogens, which finally contribute to both the reduction in rot rate and the delay in senescence of grape berries, it also delayed the senescence (browning index, weight loss rate and shedding rate of grape cluster) of grapes during postharvest storage.

## CONFLICT OF INTEREST

The authors declare that there are no conflicts of interest.

## Data Availability

Data available on request due to privacy/ethical restrictions
